# Ablation of the epithelial‐specific splicing factor Esrp1 results in ureteric branching defects and reduced nephron number

**DOI:** 10.1002/dvdy.24431

**Published:** 2016-07-28

**Authors:** Thomas W. Bebee, Sunder Sims‐Lucas, Juw Won Park, Daniel Bushnell, Benjamin Cieply, Yi Xing, Carlton M. Bates, Russ P. Carstens

**Affiliations:** ^1^ Department of Medicine (Renal Division), Perelman School of MedicineUniversity of PennsylvaniaPhiladelphiaPennsylvania; ^2^ Division of Nephrology, Department of PediatricsUniversity of Pittsburgh School of MedicinePittsburghPennsylvania; ^3^ Department of Computer Engineering and Computer Science, KBRIN Bioinformatics CoreUniversity of LouisvilleLouisvilleKentucky; ^4^ Department of Microbiology, Immunology and Molecular GeneticsUniversity of California Los AngelesLos AngelesCalifornia; ^5^ Rangos Research Center, Children's Hospital of Pittsburgh of UPMCPittsburghPennsylvania; ^6^ Department of Genetics, Perelman School of MedicineUniversity of PennsylvaniaPhiladelphiaPennsylvania

**Keywords:** alternative splicing, kidney development, fibroblast growth factor receptors, epithelial splicing regulatory proteins

## Abstract

**Background:**

Abnormalities in ureteric bud (UB) branching morphogenesis lead to congenital anomalies of the kidney and reduced nephron numbers associated with chronic kidney disease (CKD) and hypertension. Previous studies showed that the epithelial fibroblast growth factor receptor 2 (Fgfr2) IIIb splice variant supports ureteric morphogenesis in response to ligands from the metanephric mesenchyme during renal organogenesis. The epithelial‐specific splicing regulator Esrp1 is required for expression of Fgfr2‐IIIb and other epithelial‐specific splice variants. Our objective was to determine whether Esrp1 is required for normal kidney development.

**Results:**

Ablation of Esrp1 in mice, alone or together with its paralog Esrp2, was associated with reduced kidney size and increased incidence of renal aplasia. Three‐dimensional imaging showed that embryonic Esrp1 knockout (KO) kidneys had fewer ureteric tips and reduced nephron numbers. Analysis of alternative splicing in Esrp‐null ureteric epithelial cells by RNA‐Seq confirmed a splicing switch in Fgfr2 as well as numerous other transcripts.

**Conclusions:**

Our findings reveal that Esrp1‐regulated splicing in ureteric epithelial cells plays an important role in renal development. Defects in Esrp1 KO kidneys likely reflect reduced and/or absent ureteric branching, leading to decreased nephron induction secondary to incorrect Fgfr2 splicing and other splicing alterations. *Developmental Dynamics 245:991–1000, 2016*. © 2016 The Authors. Developmental Dynamics published by Wiley Periodicals, Inc. on behalf of American Association of Anatomists.

## Key findings

- Abnormalities in Ureteric bud (UB) branching morphogenesis lead to congenital anomalies of the kidney and reduced nephron numbers associated with chronic kidney disease (CKD) and hypertension.
- We investigated the consequences of ablating the epithelial‐specific splicing regulator Esrp1 on renal organogenesis and determined that Esrp1 KO mice have reduced kidney size, fewer ureteric tips, reduced nephron numbers, and increased incidence of renal aplasia.
- Analysis of alternative splicing in Esrp null ureteric epithelial cells by RNA‐Seq identified numerous alterations in splicing.
- These findings reveal that Esrp1 regulated splicing in ureteric epithelial cells plays an important role in the kidney and illustrate the importance of alternative splicing for normal renal organogenesis.

## Introduction

Developmental abnormalities of the kidney underlie a diverse array of human diseases. Kidney formation begins with outgrowth of the ureteric bud (UB) from the nephric duct in response to signals from the adjacent metanephric mesenchyme (MM). Ongoing signals from the MM drive UB growth and branching to form the renal collecting system and ureter (Nigam and Shah, 2009; Costantini and Kopan, 2010; Little et al., 2010). Reciprocal signals from the UB branch tips to the MM induce a mesenchymal‐to‐epithelial transition (MET) that gives rise to the nephron epithelia from the glomerulus to the connecting tubules. Abnormalities in UB branching morphogenesis can lead to congenital anomalies of the kidney and urinary tract (CAKUT), which are among the most common human birth defects (Schedl, 2007; Little and McMahon, 2012). In addition, abnormal ureteric branching and reduced tip numbers result in decreased nephron numbers that confer a higher risk of developing hypertension and chronic kidney disease (CKD) (Clark and Bertram, 1999; Poladia et al., 2006).

Understanding the molecular mechanisms and the gene expression networks that guide renal development is critical ultimately to therapeutically impact many kidney diseases. Numerous congenital abnormalities and genetic diseases that cause renal failure are due to mutations in key developmental genes (Schedl, 2007; Costantini, 2010). In addition, both genetic and environmental factors such as prenatal stresses and premature birth can reduce UB branching and nephron endowment (Schedl, 2007; Dressler, 2009; Costantini and Kopan, 2010; Little et al., 2010). Genetic studies in mouse models have been invaluable in identifying and characterizing key transcriptional factors, cell surface receptors, and signaling pathways that guide UB branching morphogenesis and nephron formation. For example, outgrowth of the UB occurs when MM‐derived glial cell–derived neurotrophic factor (Gdnf) interacts with the Ret receptor tyrosine kinase (RTK) and the co‐receptor Grfα1, and knockout of any of these factors in mice leads to renal agenesis or aplasia (Schuchardt et al., 1994; Pichel et al., 1996; Sanchez et al., 1996; Treanor et al., 1996; Enomoto et al., 1998).

Many studies have identified gene expression patterns and transcriptional networks in the developing kidney that are mechanistically informative. (Brunskill et al., 2008; Mugford et al., 2009; Harding et al., 2011; Thiagarajan et al., 2011; Yu et al., 2012). However, the role that alternative splicing (AS) plays in the gene expression programs and regulatory networks that underlie kidney formation are largely unknown. Recent studies have shown that nearly all mammalian multi‐exon genes produce multiple alternatively spliced mRNAs (Pan et al., 2008; Wang et al., 2008). These alternatively spliced transcripts produce protein isoforms with widely divergent functions including changes in subcellular localization, protein‐protein interactions, and post‐translational modifications (Kelemen et al., 2013). Furthermore, many AS events are tightly regulated in a cell‐type or tissue‐specific manner and at different developmental stages by RNA‐binding proteins, including cell‐ or tissue‐specific splicing factors (Chen and Manley, 2009; Nilsen and Graveley, 2010; Kalsotra and Cooper, 2011).

While the impact of AS in kidney development is unclear, it is required for expression of the proper *Fgfr2* isoform in the ureteric epithelium (Sawicka et al., 2008). Alternative splicing of mutually exclusive *Fgfr2* exons IIIb and IIIc yields receptor isoforms Fgfr2‐IIIb and Fgfr2‐IIIc in epithelial and mesenchymal cells, respectively (Zhang et al., 2006). Moreover, Fgfr2‐IIIb and Fgfr2‐IIIc isoforms have differing ligand‐binding specificities that impact development of numerous organs (Min et al., 1998; Xu et al., 1998; Zhang et al., 2006). Previously, our group has shown that mesenchymal Fgfr2‐IIIc is critical for maintenance of the developing kidney MM (Sims‐Lucas et al., 2011). In addition, isoform‐specific knockout of *Fgfr2‐IIIb* as well as its specific ligands *Fgf7* or *Fgf10* leads to reduced kidney size, nephron number, and/or renal dysgenesis (Qiao et al., 1999; Ohuchi et al., 2000; Revest et al., 2001; Michos et al., 2010). Furthermore, we showed that Hoxb7cre‐mediated conditional deletion of *Fgfr2* in the ureteric bud (where Fgfr2‐IIIb is the exclusive isoform) leads to defects in ureteric branching and secondarily to reduced nephrogenesis (Zhao et al., 2004; Sims‐Lucas et al., 2009). Others have shown mechanisms by which Fgf signaling can partially compensate for a loss of the Gdnf/Ret axis in some contexts. Deletion of the RTK inhibitor Sprouty1 (*Spry1*) led to rescue of UB branching and kidney formation in *Gdnf* or *Ret* knockout (KO) mice (Michos et al., 2010). However, in *Gdnf* ^−/−^/*Spry*1^−/−^ mice, deletion of one or both alleles of *Fgf10* caused renal aplasia. Together these findings indicate there is a requirement for mesenchymal Fgf ligands signaling via Fgfr2‐IIIb in the ureteric bud for normal UB morphogenesis. It is unclear how improper splicing of Fgfr2 would affect ureteric and overall renal development.

We discovered epithelial cell‐type‐specific splicing factors Esrp1 and Esrp2 in a genome‐wide, cell‐based screen for regulators of Fgfr2 splicing (Warzecha et al., 2009). Combined knockdown of ESRP1 and ESRP2 in human epithelial cell lines induced a complete switch from FGFR2 exon IIIb to exon IIIc splicing. Conversely, ectopic expression of Esrp1 in a mesenchymal cell line induced a switch from FGFR2‐IIIc to FGFR2‐IIIb (Warzecha et al., 2009). Thus, Esrp1/Esrp2 is the master regulator that is necessary and sufficient for the expression of the Fgfr2‐IIIb splice variant in diverse epithelial cell types. We also found that depletion of ESRP1 and ESRP2 in epithelial cell lines induced changes in splicing of numerous other transcripts that were relevant to epithelial cell biology and polarity (Warzecha et al., 2010; Dittmar et al., 2012; Yang et al., 2016). These observations hinted at a broader developmental role for the Esrps in regulating epithelial cell morphogenesis during development.

To investigate the functions of Esrp1 and Esrp2 in mammalian development, we generated mice with KO alleles for *Esrp1* and *Esrp2* (Bebee et al., 2015). Whereas Esrp2 mice were viable, *Esrp1* KO mice had 100% penetrant cleft lip associated with cleft palate (CL/P) and postnatal lethality, but no other obvious gross anatomic defects, nor reduced size or weight. In contrast, *Esrp1/Esrp2* double KO (DKO) mice demonstrated a host of more severe developmental defects. In the present study, we conducted a detailed investigation into renal developmental defects associated with KO of *Esrp1* alone, as well as *Esrp1/Esrp2* DKO. We determined that Esrp1 ablation alone induced defects in UB branching, reduced kidney size, and increased incidence of renal aplasia, which partially recapitulates the renal defects deletion of Fgfr2 in UB (Sims‐Lucas et al., 2009). We also isolated ureteric epithelial cells from Esrp1/Esrp2 DKO kidneys and identified numerous splicing switches in Esrp‐ablated cells relative to controls. Thus, the defects in UB branching morphogenesis, and thus renal morphogenesis, likely result from aberrant alternative splicing in Fgfr2 and likely other genes in the ureteric epithelium.

## Results

### Esrp1 KO Mice Exhibit Renal Hypoplasia and an Increased Incidence of Renal Aplasia

**Figure 1:**
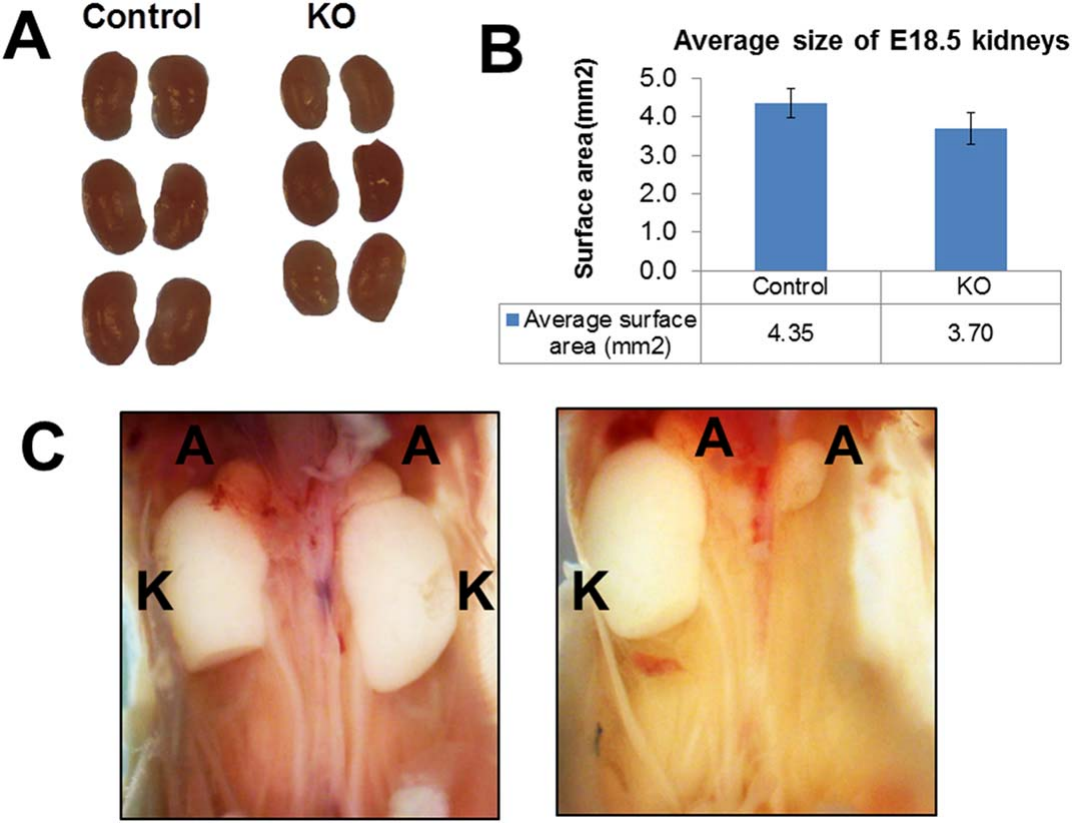
*Esrp1* KO mice have reduced kidney size compared to control littermates. **A**: Representative images of E18.5 kidneys from Control (*Esrp1* ^+/+^ and *Esrp1* ^+/‐^; N=22) and *Esrp1* KO (Esrp1^‐/‐^; N=12) mice. **B**: Graphical representation of average cross‐sectional area is shown (p‐value for difference in size = 0.00007 by two‐tailed t‐test). **C**: Example of unilateral renal agenesis in an *Esrp1^‐/‐^;Esrp2^+/‐^* E18.5 embryo (right) compared to a littermate control with both kidneys (left). A, adrenal glands; K, kidney; U, ureter.

**Figure 2:**
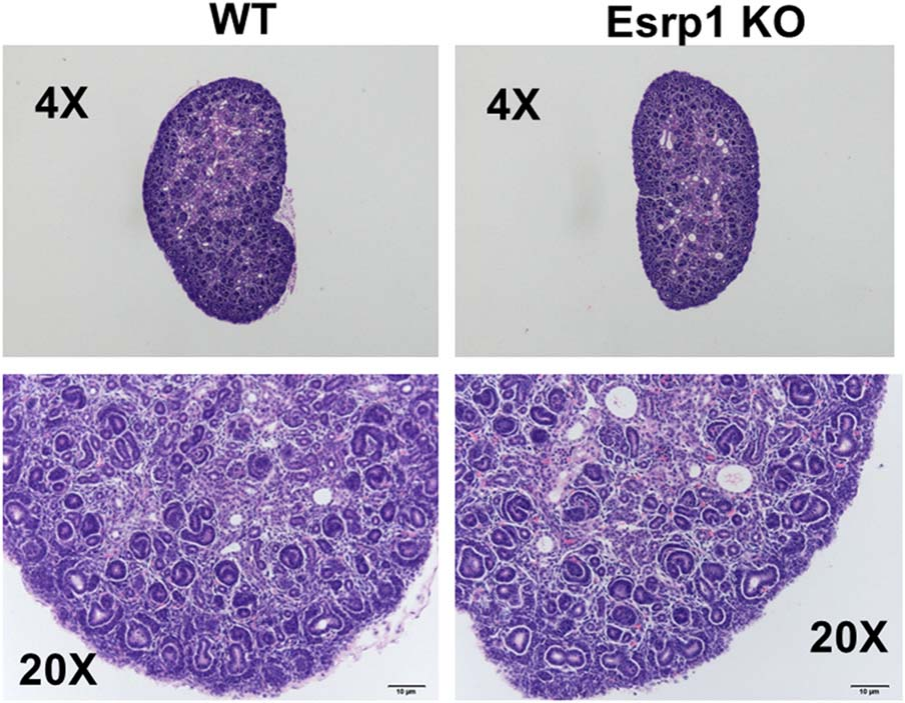
Representative H & E stained sections from wild‐type (WT) control (left panels) and Esrp1 KO kidneys (right panels) showing no apparent abnormalities in developing glomeruli or tubular structures in Esrp1 KO kidneys.

Due to postnatal lethality and frequent maternal cannibalization of *Esrp1* KO pups, we initially carried out more detailed analysis of E18.5 *Esrp1* KO embryonic kidneys. Consistent with an absence of splicing alterations in mice with at least one intact *Esrp1* allele (Bebee et al., 2015), we noted no apparent difference in the appearance or size of *Esrp1* ^*+/+*^ and *Esrp1* ^*+/‐*^ kidneys. However, *Esrp1* ^*‐/‐*^ kidneys appeared smaller and had a ∼15% decrease in mean kidney cross‐sectional area relative to control littermate kidneys (KO 4.35 ± 0.38 mm^2^, controls 3.71 ± 0.40 mm^2^, p < 0.0001; Fig. 1; as previously noted, Esrp1^‐/‐^ E18.5 embryos were not different from littermates based on weight and crown‐to‐rump length). H & E staining of tissue cross‐sections did not reveal any obvious differences in tubular epithelial cell or glomerular morphology in E18.5 mutants vs. controls (Fig. 2), consistent with renal hypoplasia in the mutants. We also assessed whether there were any redundant actions of Esrp2 with Esrp1 on kidney development by examining compound mutants. As previously reported, *Esrp1^‐/‐^;Esrp2^+/‐^* and *Esrp1^‐/‐^;Esrp2^‐/‐^* embryos and pups exhibited several significant non‐renal abnormalities, and *Esrp1/Esrp2 DKO* pups were ∼30% reduced in total size and weight (Bebee et al., 2015). However, we did not note a further apparent reduction in E18.5 kidney size when comparing *Esrp1^‐/‐^;Esrp2^+/+^* mice to *Esrp1* ^*‐/‐*^ mice that had a heterozygous or null allele for *Esrp2*. Interestingly, as we generated large numbers of mutants, we noted that a significant number of mice with *Esrp1* ^*‐/‐*^ genotypes had unilateral renal aplasia, whereas all mice with at least one wild‐type (WT) *Esrp1* allele had two kidneys, independent of the *Esrp2* allele (Table 1). In addition, we identified one *Esrp1^‐/‐^; Esrp2^‐/‐^* embryo in which both kidneys were missing. Thus, Esrp1 actions appear most critical in guiding normal kidney development without apparent redundant actions of Esrp2. The reduced kidney sizes and partially penetrant renal aplasia in *Esrp1*‐null mutants strongly suggest that alterations in the expression or splicing of Esrp1‐regulated targets lead to these kidney defects.

**Table 1:** Frequency of Renal Agenesis in Esrp1 KO E18.5 Embryos

Genotype	0 kidneys	1 kidney	2 kidneys	Frequency of renal agenesis
Controls (at least on intact Esrp1 allele)	0	0	377	0/377 (0%)
Esrp1^‐/‐^;Esrp2^+/+^	0	8	38	8/46 (17.4%)
Esrp1^‐/‐^;Esrp2^+/‐^	0	4	14	4/18 (22.2%)
Esrp1^‐/‐^;Esrp2‐/‐	1	8	59	9/68 (12.5%)

### Ablation of Esrp1 Results in Ureteric Branching Defects, Reduced Ureteric Tips, and Reduced Nephron Formation

**Figure 3:**
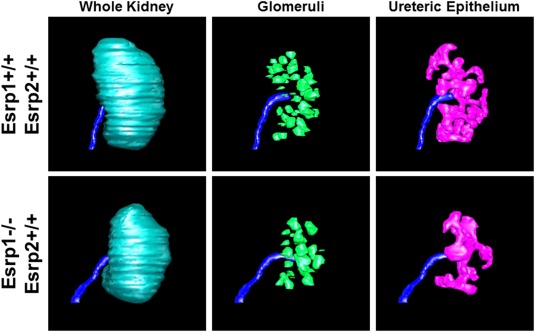
Representative 3‐D images showing reduced ureteric volume (pink) and fewer nephrons (green) in E13.5 Esrp1 KO kidneys compared to littermate controls.

To further characterize ureteric and possible secondary nephrogenesis defects *in Esrp1‐null* kidneys, we performed three‐dimensional (3‐D) reconstruction of serially sectioned E13.5 embryos to investigate branching and nephron formation in both *Esrp1* KO and *Esrp1/Esrp2* DKO kidneys. For this analysis, we compared four *Esrp1^‐/‐^:Esrp2^+/+^* (Esrp1 KO) kidneys to four WT *Esrp1^+/+^;Esrp2^+/+^* control kidneys, and four *Esrp1^‐/‐^:Esrp2^‐/‐^* (*Esrp1/Esrp2* DKO) kidneys to four Esrp1^+/+^;Esrp2^‐/‐^ control kidneys. Consistent with the analysis of E18.5 kidneys, the 3‐D reconstruction showed reduced kidney surface area by ∼25% in both *Esrp1* KO and *Esrp1/Esrp2* DKO kidneys relative to the respective controls (Fig. 3 and Table 2). In assessing ureteric volumes, we detected a trend for a reduction in mean *Esrp1* ^*‐/‐*^ ureteric volume and a significant reduction in mean *Esrp1^‐/‐^;Esrp2^‐/‐^* ureteric volume vs. littermate controls (Table 2). Moreover, relative mean ureteric volume (normalized to kidney size) and absolute mean ureteric surface area were reduced in both *Esrp1* ^*‐/‐*^ and *Esrp1^‐/‐^;Esrp2^‐/‐^* kidneys vs. littermate controls. We then skeletonized the ureteric trees and noted statistically significant decreases in mean ureteric branch and tip numbers (∼55%–60%) in both KO and DKO kidneys relative to controls. The 3‐D analysis also revealed significant reductions in *Esrp1* KO and *Esrp1;Esrp2* DKO mean developing nephron structures relative to controls that were similar to the reduced tip numbers (meaning that the nephron number loss was likely due to fewer ureteric tips inducing nephron development). Thus, these findings strongly suggested that there is a defect in developing kidneys that primarily reflects transcriptomic alterations in the ureteric lineage that leads to reduced kidney size and nephron endowment. Taken together with the increased incidence of renal aplasia in Esrp1‐null mice, these findings suggest that Esrp1 is critical for Fgfr2 splicing in the ureteric epithelium to maintain ureteric epithelial architecture.

**Table 2:** Ureteric and Nephron Measurements in Esrp KO and Control Kidneys

Measurement	Esrp1^+/+^; Esrp2^+/+^ (control)	Esrp1^‐/‐^; Esrp2^+/+^	% of controls	p‐value	Esrp1^+/+^; Esrp2^‐/‐^ (control)	Esrp1^‐/‐^; Esrp2^‐/‐^	% of controls	p‐value
Kidney surface area (× 10^5^ μm^2^)	6.07 ± 0.88	4.60 ± 0.81	76%	0.0498	5.96 ± 0.91	4.66 ± 0.45	78%	0.0426
Ureteric epithelium (UE) Volume (× 10^6^ μm^3^)	5.58 ± 1.63	3.35 ± 1.02	60%	0.0606	5.58 ± 1.06	3.21 ± 0.54	58%	0.0072
UE % of kidney (%)	12.0 ± 0.8	10.2 ± 0.6	85%	0.0090	12.3 ± 0.4	9.6 ± 0.3	78%	0.0001
UE surface area (× 10^5^ μm^2^)	4.86 ± 1.24	2.92 ± 0.79	60%	0.0391	4.70 ± 1.00	2.71 ± 0.50	58%	0.0117
Dev. glomeruli volume (× 10^6^ μm^3^)	3.54 ± 1.08	2.55 ± 0.78	72%	0.1864	3.24 ± 1.15	2.24 ± 0.60	69%	0.1742
Dev. glomeruli avg size (× 10^4^ μm^3^)	7.04 ± 1.63	7.36 ± 1.23	104%	0.7704	6.71 ± 1.04	7.59 ± 1.23	113%	0.3160
Dev. glomeruli number	49.75 ± 7.13	34.25 ± 7.27	69%	0.0227	47.25 ± 9.88	29.50 ± 5.91	62%	0.0216
Dev glomeruli % of kidney (%)	7.6 ± 1.0	7.8 ± 0.9	102%	0.8239	7.0 ± 1.2	6.7 ± 1.11	96%	0.7104
Branch number	69 ± 19.77	41 ± 0.81	59%	0.0478	85 ± 19.77	48 ± 15.06	56%	0.0130
Tip number	70 ± 18.35	43 ± 10.56	61%	0.0421	87 ± 13.67	50 ± 13.11	58%	0.0082
Branch length (μm)	7030.42 ± 1971.11	4305.75 ± 1211.58	61%	0.0567	7756.82 ± 1435.70	4530.26 ± 1332.36	58%	0.0165

### Identification of Esrp1‐regulated Splicing in Ureteric Epithelial Cells

**Figure 4:**
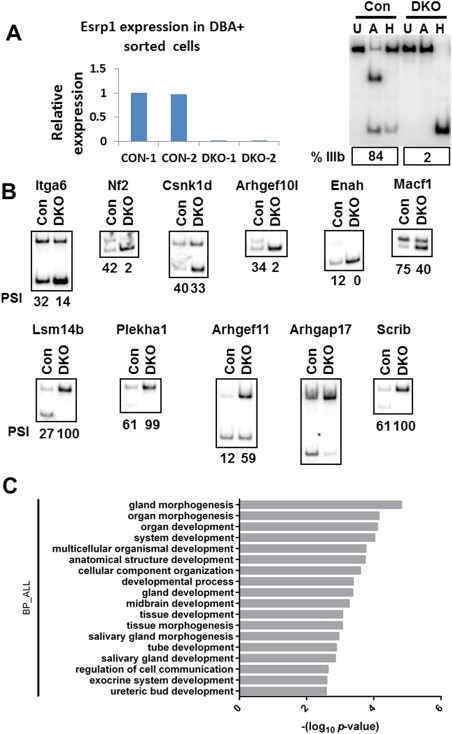
Validations of splicing changes in DBA + ureteric epithelial cells in Esrp1‐/‐;Esrp2‐/‐ DKO embryos compared to Esrp1+/+;Esrp2‐/‐ control littermates by semi‐quantitative RT‐PCR. **A**: At left is gPCR data confirming Esrp1 ablation in two Esrp1 KO sample replicates compared to control replicates. At right is validation of a nearly complete switch in Fgfr2 splicing from exon IIIb to IIIc. Note that RT‐PCR products containing exon IIIb contain a restriction site for AvaI (A), whereas those with exon IIIc have 2 HincII (H) sites that were used in restriction digests to distinguish these products. Lanes labelled U represent uncut RT‐PCR products. Quantification of exon IIIb splicing is indicated. **B**: Additional examples of validated alternative splicing switches. The quantifications for Percent Spliced In (PSI) are shown for each condition. Values for mean PSI from both replicates are shown in a tab in Table S1. **C**: GO analysis of enriched categories for genes with alternative splicing switches in DKO ureteric epithelium. Con, control; DKO, Esrp1;Esrp2 double knockout.

While previous studies indicate that there is also Esrp1 expression in distal nephron epithelial cells in addition to ureteric expression (Yu et al., 2012), the phenotypic assessments of the mutants suggest that the primary developmental defects are in ureteric morphogenesis. We note, however, that we cannot rule out defects in *Esrp1*‐null distal nephron segments that were not identified by standard H & E staining. To further investigate potential molecular mechanisms that give rise to the defects in ureteric branching, we investigated changes in AS in Esrp‐null kidneys using RNA‐Seq of ureteric epithelial cells isolated by fluorescence‐activated cell sorting (FACS). Studies from our group and others have noted that RNA‐Seq is associated with significant false negatives when used for detection of changes in alternative splicing. Thus, although we noted kidney defects in *Esrp1* KO as well as *Esrp1/Esrp2* DKO kidneys, we used *Esrp1/Esrp2* DKO embryos for splicing analysis in order to optimize detection of Esrp‐regulated splicing. Using Dolichos biflorus agglutinin coupled with Fluorescein isothiocyanate (DBA‐FITC) to label ureteric cells, we sorted DBA‐positive ureteric epithelial cells from two biological replicates each for *Esrp1^‐/‐^;Esrp2^‐/‐^* and control *Esrp1^+/+^;Esrp2^‐/‐^* E18.5 kidneys. To identify differential AS events between control and DKO samples, we used the replicate Multivariate Analysis of Transcript Splicing (rMATS) computational tool to identify differential AS events from strand‐specific RNA‐Seq data corresponding to all five basic types of AS patterns (Shen et al., 2014). For each AS event, we used both the reads mapped to the splice junctions and the reads mapped to the exon body as the input for rMATS. Differentially spliced events with an associated change in Percent Spliced In (ΔPSI or ΔΨ) of ≥ 5% and a false discovery rate of < 5% are summarized in Table S1. Of note, we identified 39 cassette exons (also called SE, for skipped exons) that underwent a change in splicing using our statistical cutoffs, seven mutually exclusive events (MXE), and two alternative 5' splice sites (A5SS). Not surprisingly, a switch in *Fgfr2* splicing was associated with the largest change in splicing of mutually exclusive exons, and this was confirmed by reverse transcription‐polymerase chain reaction (RT‐PCR) (Fig. 4A). Among the SE events, we noted several splicing changes that previously had been identified in Esrp1/Esrp2 DKO epidermis and/or Esrp‐depleted epithelial cell lines such as p120‐catenin (Ctnnd1), Cd44, Scrib, and Arhgap17. However, we also identified splicing switches that were not previously defined in Esrp‐depleted non‐ureteric epithelial cells. We used semi‐quantitative RT‐PCR to validate several additional splicing switches predicted by RNA‐Seq, as well as two additional Esrp targets (Enah and Macf1) identified in our previous studies (Fig. 4B). We confirmed splicing switches in 11 of 15 SE events tested, for which RT‐PCR was successful. We did, however, note that several of the splicing switches that included previously identified Esrp‐regulated targets were quantitatively less than observed in other contexts, which we suspect is to some degree of mesenchymal or stromal cell contamination using the DBA lectin–based sorting approach. For example, even for Fgfr2, the control pattern showed ∼20% of transcripts with the mesenchymal Fgfr2‐IIIc splice variant, consistent with baseline splicing patterns in control cells that are less epithelial than in other homogenous populations of epithelial cells previously analyzed. We thus suspect that some component of a non‐epithelial cell population was one limitation in identifying an even broader number of Esrp‐regulated targets in UB cells. In addition, the limited number of splicing changes we observed in *Esrp1;Esrp2* DKO DBA‐sorted cells reflects the limited sequencing depth obtained from the limited cell populations isolated, which is a known shortcoming of RNA‐Seq for detection of changes in alternative splicing. Nonetheless, a Gene Ontology (GO) analysis for biological processes yields enrichment of Esrp‐regulated targets for terms relevant to UB and branching morphogenesis (Fig. 4C). For example, the top three enriched terms were gland morphogenesis, organ morphogenesis, and organ development. Ureteric bud development was also among the enriched terms, based upon changes in splicing of Fgfr2, Fgfr1, and CD44. In future studies it will be useful to incorporate fluorescent reporter lines and larger numbers of replicates and expanded sequencing depth to identify an even more comprehensive set of regulated targets in the kidney, including renal tubular epithelial cells.

We also determined changes in total transcript using the FPKM metric (fragments per kilobase of exon per million fragments mapped) and identified 436 transcripts with at least a two‐fold change in gene expression between the DKO and control samples at FDR < 5% for transcripts with a minimum FPKM > 0.1 (Table S2). Interestingly, GO analysis for genes with altered expression in Esrp KO ureteric epithelium showed a high enrichment for genes involved in immune response or inflammation, including numerous genes encoding chemokines, complement components, and toll‐like receptors. Determining whether these changes in gene expression represent direct regulation of transcript stability or indirect effects requires further investigation. We note that Esrp ablation in the skin induces large‐scale changes in gene expression that we suspect are indirect due to a defect in barrier function of the epidermis. It is thus possible that some of these changes in the UB may indicate alterations in inflammatory responses, though the mechanisms for such a response are not clear. On the other hand, a previous study noted conserved Esrp1/2 binding sites in 3' untranslated regions, suggesting that Esrp1 may also regulate post‐transcriptional gene expression at the level of stability as well as splicing. Recent studies of numerous other RNA binding proteins support such multifunctional roles in post‐transcriptional regulation for an increasing number of genes (Sawicka et al., 2008).

## Discussion

This study presents a detailed view of defects in kidney development that result from ablation of the epithelial‐specific splicing factor Esrp1 and represents the first investigation of an essential splicing factor on renal organogenesis. We determined that disruption of an Esrp1‐directed epithelial splicing program is associated with several abnormalities in renal organogenesis that likely have relevance to human congenital kidney diseases that result in childhood or adult‐onset CKD and/or hypertension. First, we discovered a significantly increased risk of unilateral (and rarely bilateral) renal aplasia in many *Esrp1*‐null mice. Second, we noted that those E18.5 kidneys that did form in *Esrp1*‐null mice were hypoplastic relative to control kidneys, suggesting reduced ureteric branching and a reduced nephron mass. Third, detailed 3‐D imaging of E13.5 kidneys revealed abnormal and reduced ureteric branching and tip formation that was associated with a corresponding decrease in nephron number. Fourth, we determined that there were few, if any, redundant roles for Esrp2 with Esrp1 in ureteric morphogenesis.

The abnormalities we observed are most likely due to alterations in splicing of key Esrp target transcripts, whose functions in kidney development and other epithelial cell processes are fine‐tuned through the expression of epithelial‐specific protein isoforms, or alterations in isoform ratios. It is important to note that it is now recognized that AS events are often tightly regulated in a cell‐type or tissue‐specific manner, and at different developmental stages (Chen and Manley, 2009; Nilsen and Graveley, 2010). An emerging concept in the AS field is that, similar to transcriptional regulators, tissue‐specific splicing regulators coordinate programs of AS involving transcripts that encode proteins that function in biologically coherent pathways (Ule et al., 2005; Karni et al., 2007; Zhang et al., 2008). Thus, the definition of programs of alternative splicing directed by cell‐type‐specific splicing factors will reveal novel genes that are important for the development and functions of the specific cell types or tissues in which they are expressed. By extension, disruption of the function of these splicing factors, as well as their regulation target genes, can also result in disease. The GO analysis presented here using an Esrp‐regulated AS program in the UB is consistent with this proposition, as it consisted of a number of regulated gene transcripts known to be important for organ development, including the kidney. For example, one Esrp‐regulated target is CD44, which was previously shown to play a role in in branching morphogenesis of ureteric bud cells (Pohl et al., 2000). This example, together with Fgfr2, suggests that coordinated functions of other Esrp‐regulated genes may collectively contribute to kidney formation, and that disruption in the expression or splicing of other target genes may also be involved in kidney disease. We thus propose that some of the genes we identify here whose splicing is regulated by the Esrps in the UB may also have yet to be defined roles in kidney development, possibly through regulation of epithelial‐mesenchymal cross talk or branching morphogenesis. In addition, some of these genes may also be modifier genes associated with increased risk of renal congenital anomalies as well as chronic kidney disease. A more extensive characterization of AS in the different segments of the developing kidney, as well a more comprehensive determination of Esrp‐regulated targets in the UB and other renal epithelial cells, is expected to provide further insights into normal programs of kidney development.

While the functional consequences of the change in Fgfr2 splicing have been well characterized at the cellular level, the differential functions of most protein isoforms that result from alternative splicing of Esrp‐regulated gene transcripts remain undefined. It therefore is a challenge to dissect how alterations in splicing of defined transcripts contribute to the kidney phenotypes described here, as well as those in other organs and tissues impacted by *Esrp1* or *Esrp1/Esrp2* ablation. Nonetheless, we suspect that the loss of the epithelial Fgfr2‐IIIb isoform in ureteric epithelial cells is one of likely many key splicing changes that contribute to these phenotypes. Previous studies, for example, showed reduced kidney size in mice with isoform‐specific KO of the *Fgfr2‐IIIb* isoform as well as its specific ligands Fgf7 or Fgf10 (Qiao et al., 1999; De Moerlooze et al., 2000; Ohuchi et al., 2000; Michos et al., 2010). We also previously showed that conditional deletion of *Fgfr2* in the ureteric bud lineage was also associated with branching defects, reduced kidney size, and reduced ureteric tips and nephrons (Zhao et al., 2004; Sims‐Lucas et al., 2009). As previously noted, it has also been shown that in certain genetic contexts, Fgf10 signaling through Fgfr2‐IIIb in ureteric epithelium can compensate for impaired Gdnf/Ret signaling in renal organogenesis (Michos et al., 2010).

While the effects of Esrp loss on Fgfr2 splicing likely account for much of the ureteric and renal defects in the mutants, there are some phenotypic differences between Esrp mutants and Fgfr2 mutants. First, unlike in the *Esrp1* mutants, we did not routinely observe renal aplasia (suggesting ureteric induction defects). Second, the glomeruli in Fgfr2^UB‐/‐^ kidneys are increased in size, whereas they are unchanged in size in *Esrp1* ^*‐/‐*^ kidneys. One reason for the differences in the phenotypes is likely a key functional distinction between the Hoxb7cre deletion of Fgfr2 or the global Fgfr2 exon IIIb mutants and the Fgfr2 splice variants that result from isoform‐specific deletion of exon IIIb of Fgfr2 and Esrp ablation in our Esrp KO mice. Although deletion of exon IIIb preserves expression of Fgfr2‐IIIc in mesenchymal tissues, in epithelial cells the consequence of exon IIIb deletion is not splicing to the mutually exclusive exon IIIc, but rather the skipping of both exons. This results in a frameshift into a stop codon in the next exon; hence, the result is effectively a complete ablation of Fgfr2 expression altogether in epithelial cell types, similar to the Hoxb7cre‐mediated deletion of Fgfr2. In contrast, KO of *Esrp1/2* in epithelial cells induces a complete switch in splicing from exon IIIb to exon IIIc, resulting in ectopic expression of the normally mesenchymal Fgfr2‐IIIc isoform in epithelial cells. Therefore, while Fgfr2 in the ureteric bud and derivatives in Esrp KO mice can no longer respond to mesenchyme‐ or stromal‐derived Fgf7 or Fgf10, it can still signal in response to other Fgfs with known specificity for Fgfr2‐IIIc. Such Fgfs include Fgf9, which is expressed from the ureteric tips themselves, as well as other Fgfr2‐IIIc‐specific Fgf ligands expressed in the milieu such as Fgf8 and Fgf20 (Barak et al., 2012). Hence, it is possible that such autocrine or paracrine pathways could preserve some Fgfr2 signaling in the ureteric bud as opposed to those that effectively ablate Fgfr2 expression in the ureteric epithelium or its natural Fgf ligands. In addition, we have previously shown that ablation or depletion of Esrp1 alone (with intact Esrp2 alleles) does not induce a complete switch in *Fgfr2* splicing from exon IIIb to exon IIIc, such that there is likely some Fgfr2‐IIIb in the ureteric cells of *Esrp1* KO mice that would also be expected to preserve some Fgf7/Fgf10 responsive signaling. Finally, the high incidence of renal aplasia, which we have not observed in our *Hoxb7cre;Fgfr2* ^*Fl/Fl*^ mutants, strongly suggests that perturbations in other Esrp targets underpin some of the phenotypes seen in our Esrp mutants.

In conclusion, this study describes the role of a key splicing factor in kidney development. Further investigations into the functions of alternative splicing events regulated by the Esrps may provide further insights into the pathways and signaling programs that underlie epithelial‐mesenchymal cross talk and branching morphogenesis. These investigations begin to extend our understanding of the gene‐expression programs that affect ureteric branching and nephron formation beyond transcriptional regulation. Further studies into the functional consequences of splicing switches in some of these target genes will provide further insights into the pathways and cell‐cell interactions that are required for UB branching. It will also be of interest to use conditional KO of Esrp1/Esrp2 to further investigate the consequences of Esrp ablation in ureteric and renal tubular epithelial cells for kidney function in adult mice.

## Experimental Procedures

### Mouse Crosses

*Esrp1 KO* (*Esrp1^‐/‐^;Esrp2^+/+^*) embryos were generated in crosses of Esrp1^+/‐^, Esrp2^+/+^ mice and *Esrp1/Esrp2* DKO (*Esrp1^‐/‐^;Esrp2^‐/‐^*) embryos from crosses of Esrp1^+/‐^, Esrp2^‐/‐^ mice. Genotyping for Esrp1 and Esrp2 was performed as described (Bebee et al., 2015). Pregnant dames were euthanized by carbon dioxide, and embryos were isolated and euthanized by decapitation. All animal procedures and experiments were approved by the Institutional Animal Care and Use Committee (IACUC) at the University of Pennsylvania.

### Histologic Analysis

Kidneys were isolated from E18.5 embryos. Images of E18.5 kidneys were taken using a dissecting microscope (0.8x) along with reference scale ruler. Kidney sizes were measured using Photoshop to measure to cross‐sectional area of the kidneys. E18.5 kidneys for histological analysis were fixed in 4% paraformaldehyde overnight at 4 ºC, followed by PBS washes and transfer to 70% ethanol for processing and paraffin embedding. H & E stains were performed for gross histological analysis. Imaging of sections was performed using an Olympus BX43 microscope and cellSens software. Whole‐mount in situ hybridization (ISH) using Digoxigenin UTP‐labeled antisense RNA probes against Ret was performed on E18.5 Esrp1 KO and control dissected kidneys as described previously (Hains et al., 2008).

### 3‐dimensional Reconstructions of Kidneys

E13.5 embryos were collected for 3‐D reconstructions of Esrp1 KO and Esrp1;Esrp2 DKO kidneys and the respective littermate controls (i.e., Esrp1^+/+^, Esrp2+/ + mice were used as controls for KO embryos, and Esrp1+/+, Esrp2‐/‐ embryos were controls for DKO embryos). Four embryos for each genetic group were evaluated. Embryos were euthanized by decapitation, fixed in 4% paraformaldehyde on ice, and shipped overnight to the Bates laboratory for 3‐D reconstruction as described (Sims‐Lucas et al., 2009).

### Isolation of DBA Lectin–positive Ureteric Epithelium

Ureteric epithelium (UE) from E18.5 control (Esrp1^+/+^;Esrp2^‐/‐^) and DKO (Esrp1^‐/‐^;Esrp2^‐/‐^) embryos was isolated from E18.5 embryos using FITC‐DBA lectin and FACS. E18.5 embryonic kidney pairs isolated from individual embryos were placed in PBS on ice, then transferred into 500 μl of 0.03% collagenase (Sigma, Cat#C1889) in PBS for 10 minutes at 37 ºC. Samples were transferred to ice, and the kidneys were dissociated by trituration using an 18‐gauge needle with 8–10 passes, followed by a 25‐gauge needle for 8–10 passes to homogenize to a single‐cell slurry. Cells were transferred to a 15‐ml falcon tube containing 4–5 ml 2% fetal bovine serum (FBS) in PBS to inhibit collagenase. The cells were pelleted for five minutes at 400 x g (4 ºC) and washed three times in 5 ml of 2% FBS. The final cell pellet was resuspended in 60 μl of 2% FBS in PBS and transferred to an Eppendorf tube. Cell count and viability were measured using 5 μl of cells + 5 μl trypan blue and a Hemocytometer; 5 μl of the remaining cell volume from each sample was pooled to serve as an unstained control. The remaining 50 μl was processed for labeling with FITC‐DBA lectin. The cells were pelleted at 400 x g for five minutes and stained in a fresh 50 μl of staining solution on ice and in the dark with DBA‐FITC (Vector Labs, Cat#FL‐1031) 1:10 diluted in 2% FBS in PBS for 20 minutes. The cells were washed in 1 ml of 2% FBS in PBS and centrifuged at 400 x g (4 ºC) for five minutes. The cell pellet was resuspended in 300–400 μl of 2% FBS in PBS for FACS. GFP‐positive gated cells were isolated using the unstained cells as a negative control. GFP + cells were collected directly into 500 μl of TRIzol (ThermoFisher) and snap‐frozen on dry ice and stored at ‐80 ºC.

### RNA Sequencing and Data Analysis

Total RNA from FACS‐sorted DBA + from control E18.5 control and DKO UE in TRIzol was isolated according to the manufacturer's protocol and resuspended in 10mM Tris‐Cl pH 8.0. Each biological replicate (n = 2 per genetic group) was comprised of three separate, individual embryos isolations. Each pooled biological replicate was made using 66.6 ng of total RNA, for a total of 200 ng. The pooled 200‐ng samples were used for poly A selected RNA‐Seq library preparation using the NEBNext® Ultra™ Directional RNA Library Prep Kit for Illumina® (mRNA) (New England Biolabs) [products: NEBNext® Poly(A) mRNA Magnetic Isolation Module (E7490S) and NEBNext® Ultra™ Directional RNA Library Prep Kit for Illumina® (E7420S)]. Biological replicates (n = 2 per genetic group) were individually bar‐coded, pooled, and sequenced in a single lane of a HiSeq 2000 for 100 × 2 bp paired‐end RNA‐Seq at the Penn Next Generation Sequencing Core (NGSC) Facility. RNA‐Seq reads were mapped to the mouse genome (mm10) and a data set of all possible exon‐exon junction reads as previously described (Bebee et al., 2015). The RNA‐Seq data has been deposited into the NCBI Gene Expression Omnibus (GEO) under the accession number GSE81716. RNA‐Seq reads were mapped to the mouse genome (mm10) and transcriptome (Ensembl, release 72) as previously described (Bebee et al., 2015). Differences in gene expression were determined using the FPKM metric at FDR < 5%, > two‐fold difference in gene expression based on average FPKM, and minFPKM > 0.1. To identify differential AS events between the control and DKO samples, we used rMATS to identify differential AS events from strand‐specific RNA‐Seq data corresponding to all five basic types of AS patterns as previously described (Shen et al., 2014; Bebee et al., 2015). AS events with an associated change in Percent Spliced In (ΔPSI or ΔΨ) of these events were identified at an FDR < 5% and |ΔΨ| ≥ 5%.

### RT‐PCR and Real‐time RT‐PCR

For synthesis of cDNA, 100 ng of pooled total RNA representing each biological replicate used in the RNA‐Seq experiment was used for random hexamer‐primed M‐MLV reverse transcriptase (Promega). Real‐time analysis of Esrp expression was evaluated using TaqMan probes for Esrp1 (Mm01220936_g1) and Gapdh (Mm99999915_g1) (LifeTechnologies) using a 7500 Fast Real‐Time machine (AppliedBiosystems). Semi‐quantitative radioactive RT‐PCR products were separated on 5% PAGE gels, dried and exposed on phosphorscreens, scanned on a Typhoon FLA 9500, and quantified using ImageQuant TL, version 7.0. Splicing ratios are represented as Percent Spliced In (PSI) of the alternative exon for cassette exons, and were normalized to RT‐PCR product sizes. The mean inclusion levels of the indicated exons for all validated splicing events are indicated in a summary table of Esrp‐regulated splicing events represented as mean values derived from each KO and control replicate (see tab in Table S1). Quantification of exon IIIb and IIIc for Fgfr1 and Fgfr2 required restriction enzyme specific to discriminate the two isoforms. Fgfr2 PCR products were digested with AvaI (IIIb) or HincII (IIIc). Fgfr1 products were digested with BstXI (IIIb) and HincII (IIIc) (all restriction digestions were performed according to NEB guidelines at 5U/digestion). Primer sequences are available on request. Graphical representation of PSI for IIIb inclusion was calculated as the ratio of IIIb/(IIIb + IIIc).

## Supporting information

Additional supporting information may be found in the online version of this article.

Supporting Information Table S1

Supporting Information Table S2

## References

[dvdy24431-bib-0001] Barak H , Huh SH , Chen S , Jeanpierre C , Martinovic J , Parisot M , Bole‐Feysot C , Nitschke P , Salomon R , Antignac C , Ornitz DM , Kopan R . 2012 FGF9 and FGF20 maintain the stemness of nephron progenitors in mice and man. Dev Cell 22:1191–1207. 22698282 10.1016/j.devcel.2012.04.018 PMC3376351

[dvdy24431-bib-0002] Bebee TW , Park JW , Sheridan KI , Warzecha CC , Cieply BW , Rohacek AM , Xing Y , Carstens RP . 2015 The splicing regulators Esrp1 and Esrp2 direct an epithelial splicing program essential for mammalian development. Elife 4. 10.7554/eLife.08954 PMC4566030 26371508

[dvdy24431-bib-0003] Brunskill EW , Aronow BJ , Georgas K , Rumballe B , Valerius MT , Aronow J , Kaimal V , Jegga AG , Yu J , Grimmond S , McMahon AP , Patterson LT , Little MH , Potter SS . 2008 Atlas of gene expression in the developing kidney at microanatomic resolution. Dev Cell 15:781–791. 19000842 10.1016/j.devcel.2008.09.007 PMC2653061

[dvdy24431-bib-0004] Chen M , Manley JL . 2009 Mechanisms of alternative splicing regulation: insights from molecular and genomics approaches. Nat Rev Mol Cell Biol 10:741–754. 19773805 10.1038/nrm2777 PMC2958924

[dvdy24431-bib-0005] Clark AT , Bertram JF . 1999 Molecular regulation of nephron endowment. Am J Physiol 276:F485–497. 10198407 10.1152/ajprenal.1999.276.4.F485

[dvdy24431-bib-0006] Costantini F . 2010 GDNF/Ret signaling and renal branching morphogenesis: From mesenchymal signals to epithelial cell behaviors. Organogenesis 6:252–262. 21220964 10.4161/org.6.4.12680 PMC3055651

[dvdy24431-bib-0007] Costantini F , Kopan R . 2010 Patterning a complex organ: branching morphogenesis and nephron segmentation in kidney development. Dev Cell 18:698–712. 20493806 10.1016/j.devcel.2010.04.008 PMC2883254

[dvdy24431-bib-0008] De Moerlooze L , Spencer‐Dene B , Revest J , Hajihosseini M , Rosewell I , Dickson C . 2000 An important role for the IIIb isoform of fibroblast growth factor receptor 2 (FGFR2) in mesenchymal‐epithelial signalling during mouse organogenesis. Development 127:483–492. 10631169 10.1242/dev.127.3.483

[dvdy24431-bib-0009] Dittmar KA , Jiang P , Park JW , Amirikian K , Wan J , Shen S , Xing Y , Carstens RP . 2012 Genome‐Wide Determination of a Broad ESRP‐Regulated Posttranscriptional Network by High‐Throughput Sequencing. Mol Cell Biol 32:1468–1482. 22354987 10.1128/MCB.06536-11 PMC3318588

[dvdy24431-bib-0010] Dressler GR . 2009 Advances in early kidney specification, development and patterning. Development 136:3863–3874. 19906853 10.1242/dev.034876 PMC2778737

[dvdy24431-bib-0011] Enomoto H , Araki T , Jackman A , Heuckeroth RO , Snider WD , Johnson EM Jr , Milbrandt J . 1998 GFR alpha1‐deficient mice have deficits in the enteric nervous system and kidneys. Neuron 21:317–324. 9728913 10.1016/s0896-6273(00)80541-3

[dvdy24431-bib-0012] Hains D , Sims‐Lucas S , Kish K , Saha M , McHugh K , Bates CM . 2008 Role of fibroblast growth factor receptor 2 in kidney mesenchyme. Pediatr Res 64:592–598. 18670373 10.1203/PDR.0b013e318187cc12 PMC2647852

[dvdy24431-bib-0013] Harding SD , Armit C , Armstrong J , Brennan J , Cheng Y , Haggarty B , Houghton D , Lloyd‐MacGilp S , Pi X , Roochun Y , Sharghi M , Tindal C , McMahon AP , Gottesman B , Little MH , Georgas K , Aronow BJ , Potter SS , Brunskill EW , Southard‐Smith EM , Mendelsohn C , Baldock RA , Davies JA , Davidson D . 2011 The GUDMAP database—an online resource for genitourinary research. Development 138:2845–2853. 21652655 10.1242/dev.063594 PMC3188593

[dvdy24431-bib-0014] Kalsotra A , Cooper TA . 2011 Functional consequences of developmentally regulated alternative splicing. Nat Rev Genet 12:715–729. 21921927 10.1038/nrg3052 PMC3321218

[dvdy24431-bib-0015] Karni R , de Stanchina E , Lowe SW , Sinha R , Mu D , Krainer AR . 2007 The gene encoding the splicing factor SF2/ASF is a proto‐oncogene. Nat Struct Mol Biol 14:185–193. 17310252 10.1038/nsmb1209 PMC4595851

[dvdy24431-bib-0016] Kelemen O , Convertini P , Zhang Z , Wen Y , Shen M , Falaleeva M , Stamm S . 2013 Function of alternative splicing. Gene 514:1–30. 22909801 10.1016/j.gene.2012.07.083 PMC5632952

[dvdy24431-bib-0017] Little M , Georgas K , Pennisi D , Wilkinson L . 2010 Kidney development: two tales of tubulogenesis. Curr Top Dev Biol 90:193–229. 20691850 10.1016/S0070-2153(10)90005-7

[dvdy24431-bib-0018] Little MH , McMahon AP . 2012 Mammalian kidney development: principles, progress, and projections. Cold Spring Harb Perspect Biol 4. 10.1101/cshperspect.a008300 PMC3331696 22550230

[dvdy24431-bib-0020] Michos O , Cebrian C , Hyink D , Grieshammer U , Williams L , D'Agati V , Licht JD , Martin GR , Costantini F . 2010 Kidney development in the absence of Gdnf and Spry1 requires Fgf10. PLoS Genet 6:e1000809. 20084103 10.1371/journal.pgen.1000809 PMC2797609

[dvdy24431-bib-0021] Min H , Danilenko DM , Scully SA , Bolon B , Ring BD , Tarpley JE , DeRose M , Simonet WS . 1998 Fgf‐10 is required for both limb and lung development and exhibits striking functional similarity to Drosophila branchless. Genes Dev 12:3156–3161. 9784490 10.1101/gad.12.20.3156 PMC317210

[dvdy24431-bib-0022] Mugford JW , Yu J , Kobayashi A , McMahon AP . 2009 High‐resolution gene expression analysis of the developing mouse kidney defines novel cellular compartments within the nephron progenitor population. Dev Biol 333:312–323. 19591821 10.1016/j.ydbio.2009.06.043 PMC2748313

[dvdy24431-bib-0023] Nigam SK , Shah MM . 2009 How does the ureteric bud branch? J Am Soc Nephrol 20:1465–1469. 19056872 10.1681/ASN.2008020132

[dvdy24431-bib-0024] Nilsen TW , Graveley BR . 2010 Expansion of the eukaryotic proteome by alternative splicing. Nature 463:457–463. 20110989 10.1038/nature08909 PMC3443858

[dvdy24431-bib-0025] Ohuchi H , Hori Y , Yamasaki M , Harada H , Sekine K , Kato S , Itoh N . 2000 FGF10 acts as a major ligand for FGF receptor 2 IIIb in mouse multi‐organ development. Biochem Biophys Res Commun 277:643–649. 11062007 10.1006/bbrc.2000.3721

[dvdy24431-bib-0026] Pan Q , Shai O , Lee LJ , Frey BJ , Blencowe BJ . 2008 Deep surveying of alternative splicing complexity in the human transcriptome by high‐throughput sequencing. Nat Genet 40:1413–1415. 18978789 10.1038/ng.259

[dvdy24431-bib-0027] Pichel JG , Shen L , Sheng HZ , Granholm AC , Drago J , Grinberg A , Lee EJ , Huang SP , Saarma M , Hoffer BJ , Sariola H , Westphal H . 1996 Defects in enteric innervation and kidney development in mice lacking GDNF. Nature 382:73–76. 8657307 10.1038/382073a0

[dvdy24431-bib-0028] Pohl M , Sakurai H , Stuart RO , Nigam SK . 2000 Role of hyaluronan and CD44 in in vitro branching morphogenesis of ureteric bud cells. Dev Biol 224:312–325. 10926769 10.1006/dbio.2000.9783

[dvdy24431-bib-0029] Poladia DP , Kish K , Kutay B , Bauer J , Baum M , Bates CM . 2006 Link between reduced nephron number and hypertension: studies in a mutant mouse model. Pediatr Res 59:489–493. 16549517 10.1203/01.pdr.0000202764.02295.45

[dvdy24431-bib-0030] Qiao J , Uzzo R , Obara‐Ishihara T , Degenstein L , Fuchs E , Herzlinger D . 1999 FGF‐7 modulates ureteric bud growth and nephron number in the developing kidney. Development 126:547–554. 9876183 10.1242/dev.126.3.547

[dvdy24431-bib-0031] Revest JM , Spencer‐Dene B , Kerr K , De Moerlooze L , Rosewell I , Dickson C . 2001 Fibroblast growth factor receptor 2‐IIIb acts upstream of Shh and Fgf4 and is required for limb bud maintenance but not for the induction of Fgf8, Fgf10, Msx1, or Bmp4. Dev Biol 231:47–62. 11180951 10.1006/dbio.2000.0144

[dvdy24431-bib-0032] Sanchez MP , Silos‐Santiago I , Frisen J , He B , Lira SA , Barbacid M . 1996 Renal agenesis and the absence of enteric neurons in mice lacking GDNF. Nature 382:70–73. 8657306 10.1038/382070a0

[dvdy24431-bib-0033] Sawicka K , Bushell M , Spriggs KA , Willis AE . 2008 Polypyrimidine‐tract‐binding protein: a multifunctional RNA‐binding protein. Biochem Soc Trans 36:641–647. 18631133 10.1042/BST0360641

[dvdy24431-bib-0034] Schedl A . 2007 Renal abnormalities and their developmental origin. Nat Rev Genet 8:791–802. 17878895 10.1038/nrg2205

[dvdy24431-bib-0035] Schuchardt A , D'Agati V , Larsson‐Blomberg L , Costantini F , Pachnis V . 1994 Defects in the kidney and enteric nervous system of mice lacking the tyrosine kinase receptor Ret. Nature 367:380–383. 8114940 10.1038/367380a0

[dvdy24431-bib-0036] Shen S , Park JW , Lu ZX , Lin L , Henry MD , Wu YN , Zhou Q , Xing Y . 2014 rMATS: robust and flexible detection of differential alternative splicing from replicate RNA‐Seq data. Proc Natl Acad Sci U S A 111:E5593–5601. 25480548 10.1073/pnas.1419161111 PMC4280593

[dvdy24431-bib-0037] Sims‐Lucas S , Argyropoulos C , Kish K , McHugh K , Bertram JF , Quigley R , Bates CM . 2009a Three‐dimensional imaging reveals ureteric and mesenchymal defects in Fgfr2‐mutant kidneys. J Am Soc Nephrol 20:2525–2533. 19833900 10.1681/ASN.2009050532 PMC2794230

[dvdy24431-bib-0038] Sims‐Lucas S , Cusack B , Baust J , Eswarakumar VP , Masatoshi H , Takeuchi A , Bates CM . 2011 Fgfr1 and the IIIc isoform of Fgfr2 play critical roles in the metanephric mesenchyme mediating early inductive events in kidney development. Dev Dyn 240:240–249. 21128305 10.1002/dvdy.22501 PMC3093196

[dvdy24431-bib-0039] Thiagarajan RD , Cloonan N , Gardiner BB , Mercer TR , Kolle G , Nourbakhsh E , Wani S , Tang D , Krishnan K , Georgas KM , Rumballe BA , Chiu HS , Steen JA , Mattick JS , Little MH , Grimmond SM . 2011 Refining transcriptional programs in kidney development by integration of deep RNA‐sequencing and array‐based spatial profiling. BMC Genomics 12:441. 21888672 10.1186/1471-2164-12-441 PMC3180702

[dvdy24431-bib-0040] Treanor JJ , Goodman L , de Sauvage F , Stone DM , Poulsen KT , Beck CD , Gray C , Armanini MP , Pollock RA , Hefti F , Phillips HS , Goddard A , Moore MW , Buj‐Bello A , Davies AM , Asai N , Takahashi M , Vandlen R , Henderson CE , Rosenthal A . 1996 Characterization of a multicomponent receptor for GDNF. Nature 382:80–83. 8657309 10.1038/382080a0

[dvdy24431-bib-0041] Ule J , Ule A , Spencer J , Williams A , Hu JS , Cline M , Wang H , Clark T , Fraser C , Ruggiu M , Zeeberg BR , Kane D , Weinstein JN , Blume J , Darnell RB . 2005 Nova regulates brain‐specific splicing to shape the synapse. Nat Genet 37:844–852. 16041372 10.1038/ng1610

[dvdy24431-bib-0042] Wang ET , Sandberg R , Luo S , Khrebtukova I , Zhang L , Mayr C , Kingsmore SF , Schroth GP , Burge CB . 2008 Alternative isoform regulation in human tissue transcriptomes. Nature 456:470–476. 18978772 10.1038/nature07509 PMC2593745

[dvdy24431-bib-0043] Warzecha CC , Jiang P , Amirikian K , Dittmar KA , Lu H , Shen S , Guo W , Xing Y , Carstens RP . 2010 An ESRP‐regulated splicing programme is abrogated during the epithelial‐mesenchymal transition. EMBO J 29:3286–3300. 20711167 10.1038/emboj.2010.195 PMC2957203

[dvdy24431-bib-0044] Warzecha CC , Sato TK , Nabet B , Hogenesch JB , Carstens RP . 2009 ESRP1 and ESRP2 are epithelial cell‐type‐specific regulators of FGFR2 splicing. Mol Cell 33:591–601. 19285943 10.1016/j.molcel.2009.01.025 PMC2702247

[dvdy24431-bib-0045] Xu X , Weinstein M , Li C , Naski M , Cohen RI , Ornitz DM , Leder P , Deng C . 1998 Fibroblast growth factor receptor 2 (FGFR2)‐mediated reciprocal regulation loop between FGF8 and FGF10 is essential for limb induction. Development 125:753–765. 9435295 10.1242/dev.125.4.753

[dvdy24431-bib-0046] Yang Y , Park JW , Bebee TW , Warzecha CC , Guo Y , Shang X , Xing Y , Carstens RP . 2016 Determination of a Comprehensive Alternative Splicing Regulatory Network and Combinatorial Regulation by Key Factors during the Epithelial‐to‐Mesenchymal Transition. Mol Cell Biol 36:1704–1719. 27044866 10.1128/MCB.00019-16 PMC4959312

[dvdy24431-bib-0047] Yu J , Valerius MT , Duah M , Staser K , Hansard JK , Guo JJ , McMahon J , Vaughan J , Faria D , Georgas K , Rumballe B , Ren Q , Krautzberger AM , Junker JP , Thiagarajan RD , Machanick P , Gray PA , van Oudenaarden A , Rowitch DH , Stiles CD , Ma Q , Grimmond SM , Bailey TL , Little MH , McMahon AP . 2012 Identification of molecular compartments and genetic circuitry in the developing mammalian kidney. Development 139:1863–1873. 22510988 10.1242/dev.074005 PMC3328182

[dvdy24431-bib-0048] Zhang C , Zhang Z , Castle J , Sun S , Johnson J , Krainer AR , Zhang MQ . 2008 Defining the regulatory network of the tissue‐specific splicing factors Fox‐1 and Fox‐2. Genes Dev 22:2550–2563. 18794351 10.1101/gad.1703108 PMC2546699

[dvdy24431-bib-0049] Zhang X , Ibrahimi OA , Olsen SK , Umemori H , Mohammadi M , Ornitz DM . 2006 Receptor specificity of the fibroblast growth factor family. The complete mammalian FGF family. J Biol Chem 281:15694–15700. 16597617 10.1074/jbc.M601252200 PMC2080618

[dvdy24431-bib-0050] Zhao H , Kegg H , Grady S , Truong HT , Robinson ML , Baum M , Bates CM . 2004 Role of fibroblast growth factor receptors 1 and 2 in the ureteric bud. Dev Biol 276:403–415. 15581874 10.1016/j.ydbio.2004.09.002 PMC4131686

